# Combinatory repair strategy to promote axon regeneration and functional recovery after chronic spinal cord injury

**DOI:** 10.1038/s41598-017-09432-6

**Published:** 2017-08-21

**Authors:** Marc A. DePaul, Ching-Yi Lin, Jerry Silver, Yu-Shang Lee

**Affiliations:** 1 Case Western Reserve Univ., Dept. of Neurosciences, 10900 Euclid Ave., SOM E654, Cleveland, OH 44106 USA; 2 Department of Neurosciences, Lerner Research Institute, Cleveland Clinic, Cleveland, Ohio, 44195 USA

## Abstract

Eight weeks post contusive spinal cord injury, we built a peripheral nerve graft bridge (PNG) through the cystic cavity and treated the graft/host interface with acidic fibroblast growth factor (aFGF) and chondroitinase ABC (ChABC). This combinatorial strategy remarkably enhanced integration between host astrocytes and graft Schwann cells, allowing for robust growth, especially of catecholaminergic axons, through the graft and back into the distal spinal cord. In the absence of aFGF+ChABC fewer catecholaminergic axons entered the graft, no axons exited, and Schwann cells and astrocytes failed to integrate. In sharp contrast with the acutely bridge-repaired cord, in the chronically repaired cord only low levels of serotonergic axons regenerated into the graft, with no evidence of re-entry back into the spinal cord. The failure of axons to regenerate was strongly correlated with a dramatic increase of SOCS3 expression. While regeneration was more limited overall than at acute stages, our combinatorial strategy in the chronically injured animals prevented a decline in locomotor behavior and bladder physiology outcomes associated with an invasive repair strategy. These results indicate that PNG+aFGF+ChABC treatment of the chronically contused spinal cord can provide a permissive substrate for the regeneration of certain neuronal populations that retain a growth potential over time, and lead to functional improvements.

## Introduction

The vast majority of research into treatments for spinal cord injury (SCI) focuses predominantly on the acute stages, with far fewer exploring therapies that target the chronically injured cord. Many interventions, including cellular transplantation, promote functional recovery when administered in the acute stages of SCI but ultimately fail when applied chronically^1, 2^. Repairing a long-standing damaged cord has proven to be a far more challenging goal, as there is dense and well-formed glial scarring around the lesion epicenter and increases in the perineuronal net all along the neuraxis, with both containing abundant levels of inhibitory chondroitin sulfate proteoglycans (CSPGs)^3, 4^. Unlike acute SCI, chronically injured neurons exist in a persistent dystrophic state having become entrapped in the glial scar and by forming strong synaptic-like connections with CSPG-expressing NG2 cells^5^. In addition, enduring axotomized neurons express low levels of regeneration-associated genes^6, 7^, resulting in reduced intrinsic regeneration potential. Due to the permanent loss of ascending and descending axonal pathways, chronic demyelination, and the lack of substantial axonal regeneration and plasticity, spontaneous recovery rarely, if ever, occurs in the chronically injured spinal cord^8–10^.

Certain populations of chronically injured central nervous system (CNS) neurons do, however, retain the ability to regenerate in a highly permissive environment, such as into a graft of support cells made of fetal tissue, peripheral nerves, or olfactory ensheathing cells^11–17^. Axonal growth into and along the graft can occur, but most axons fail to exit its distal end, thereby limiting functional recovery. Combining a graft with exogenous neurotrophins, eliminating inhibitory molecules, and/or supplying factors that improve the graft-cord interface can enhance the abundance of axons entering and exiting an acutely applied graft. For example, digestion of CSPG side chains with the bacterial enzyme chondroitinase ABC (ChABC) augments axonal regeneration out of a peripheral nerve graft (PNG) and facilitates recovery^18, 19^. The digestion of CSPGs also promotes the migration and integration of Schwann cells from the PNG into the host spinal cord^20^, and allows astrocytes to align tangentially along the injury site^21^. Growth factors such as GDNF, BDNF, NT-3, CNTF, or aFGF applied acutely have been shown to promote axon regeneration into and out of a graft^22–25^. Of particular interest is aFGF, which is essential for the formation of glial bridges upon which regenerating axons cross a lesion to repair injured spinal cords in lower vertebrates^26^. Mammalian astrocytes treated with aFGF display decreased levels of activation^27^ and undergo a morphological change from an activated stellate morphology to an elongated bipolar shape, encouraging integration between the PNG and spinal cord^26, 28^. In a complete transection SCI model, acute repair of the injury through the tripartite combination of PNG, aFGF, and ChABC promoted long-distance axonal regeneration through the PNG and back into the distal cord, restoring supraspinal control of bladder function. Removal of a single factor diminished axonal regeneration and recovery^28^.

In this study, we investigated a triple combinatorial PNG+aFGF+ChABC treatment paradigm in a more clinically relevant chronic contusion model, where a thick and mature glial scar surrounds a fluid-filled cystic cavity. Within the cavity, we constructed a bridge consisting of multiple intercostal nerve autografts supplemented with ChABC and aFGF. We report here that such a bridging strategy in a chronically contused spinal cord promotes significant integration between the graft and host tissue, allowing catecholaminergic but, interestingly, not serotonergic axons to regenerate beyond the lesion site. While regeneration was more limited than at acute stages, we observed some improvements in locomotor behavior and bladder physiology in chronically injured animals.

## Results

### Experimental design

**Figure 1:**
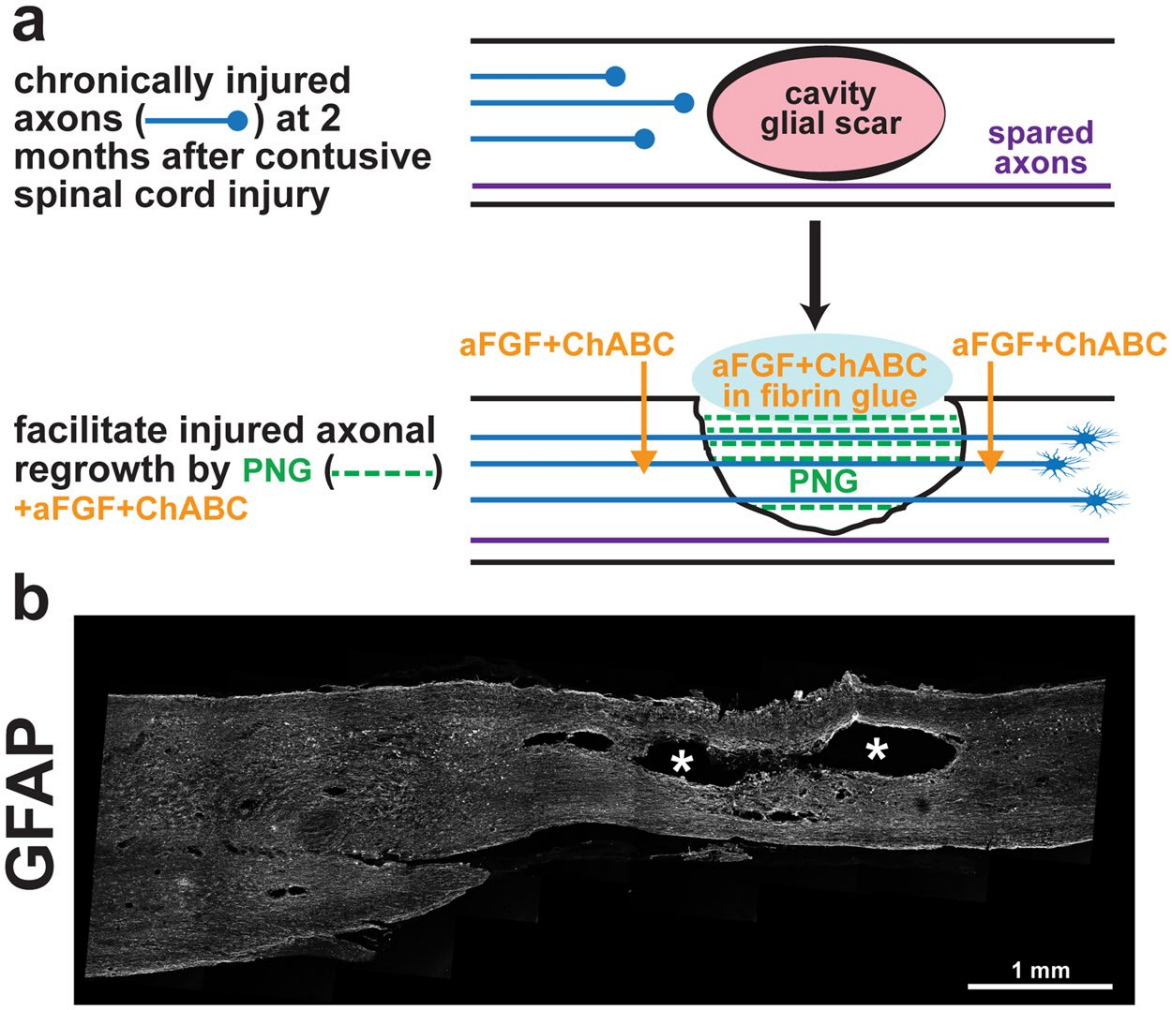
Surgical intervention to bridge the gap after chronic contusive SCI. (**a**) Schematic diagram illustrating the repair strategy. At two months post-injury, a cystic cavity forms at the injury site (pink oval, top drawing). For repair surgery, the cavity is exposed via the dorsal cord surface, scar tissue lining the cavity is gently removed, and several autologous peripheral nerve segments (green dotted lines, bottom drawing) are longitudinally placed in the cavity to span the lesion. aFGF+ChABC (orange) is injected rostrally, caudally, and at the sides of the spinal cord adjacent to the graft. A glue made of aFGF, ChABC, and fibrin is used to stabilize the graft (blue oval, bottom drawing). (**b**) GFAP-stained spinal cord two months post-contusion. Note the large cavity in the epicenter of the injured site, as indicated by*.

Contusive injury is the most common form of SCI affecting humans^29^. We modeled this injury in rodents using the Infinite Horizon device by delivering a controlled impact (250 kDyne) to the spinal cord at thoracic level 8 (T8). Over the course of weeks, a fluid-filled, scar-encased, cystic cavity forms at the injury site (Fig. 1a,b). Eight weeks following the initial injury, animals were randomly placed into one of three groups: PNG, aFGF+ChABC, or PNG+aFGF+ChABC. In all animals, a gentle incision (~2 to 3 mm) was made in the dorsal part of the spinal cord near the injury site to expose the cavity. Mature scar tissue was gently removed manually in multiple pieces from the cavity wall. One cohort of animals received injections of aFGF+ChABC with no PNG and another received aFGF+ChABC with a PNG. A third group received a PNG with only saline injections. In the two PNG groups, two to four intercostal nerve segments were collected, soaked in ChABC, and then placed in the lesion cavity to create an autologous PNG bridge (Fig. 1a).

### Catecholaminergic axons, but not serotonergic axons, regenerate through the peripheral nerve graft and back into the distal cord

**Figure 2:**
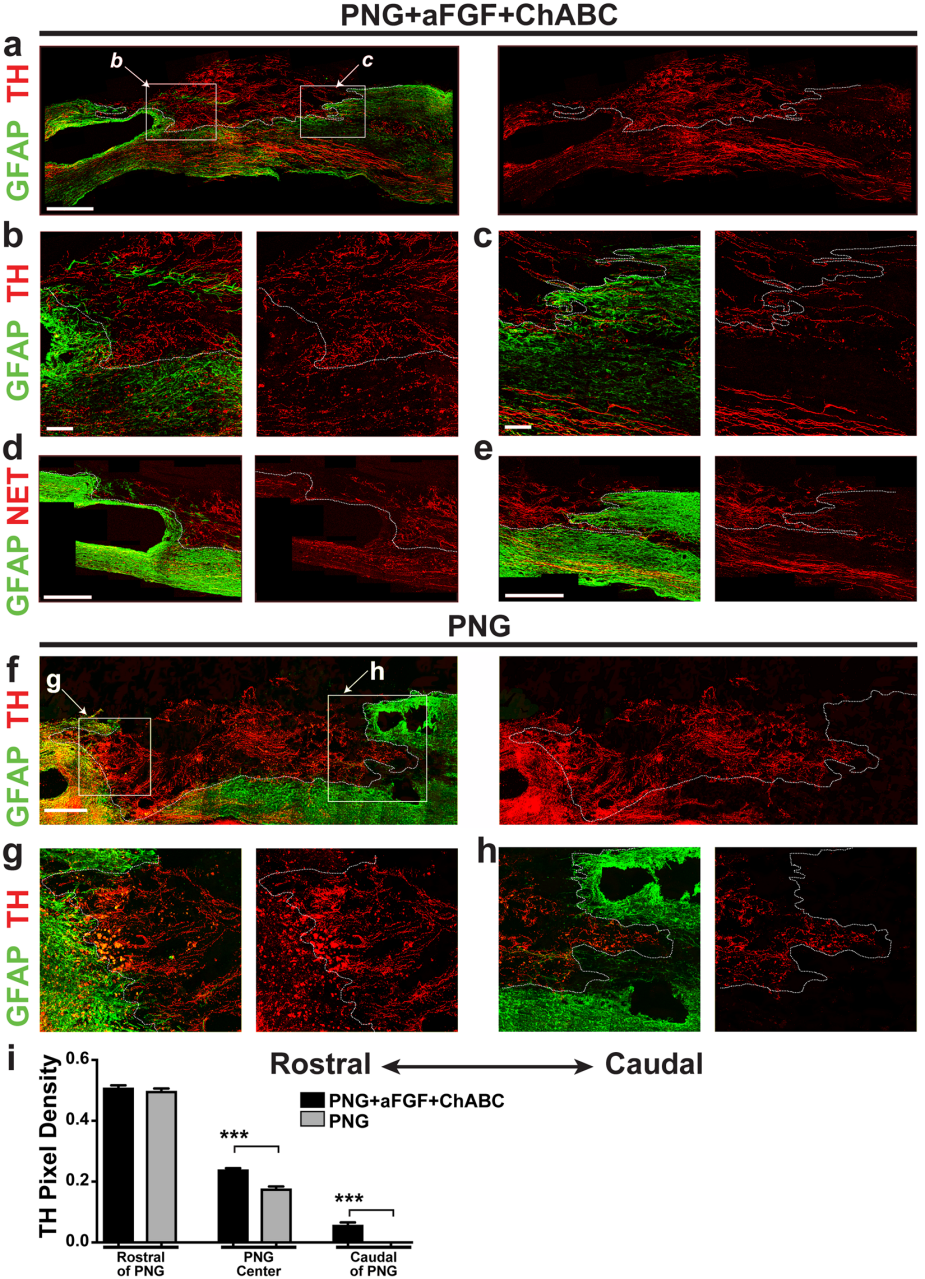
TH+ nerve fibers regenerate through a PNG and re-enter the distal spinal cord following PNG+aFGF+ChABC treatment. (**a**–**e**) Images are from PNG+aFGF+ChABC-treated animals. The dotted line in each panel delineates the interface between the graft and spinal cord. (**a**) A representative low-magnification confocal image of a sagittal section with GFAP (green) and TH (red) staining displaying the overall anatomy of the PNG, spinal cord, and regenerating TH+ axons. Scale bar, 500 µm. (**b**) High-magnification confocal image of the rostral cord/PNG interface boxed in a. Large densities of TH+ axons (red) enter the PNG (GFAP-negative area) from the rostral end of the spinal cord (green). Scale bar, 100 µm. (**c**) High-magnification confocal image of caudal PNG/cord interface boxed in a. TH+ axons (red) grow through the PNG and cross the PNG-spinal cord interface back into the caudal spinal cord (green). Scale bar, 100 µm. (**d**) High-magnification confocal image showing NET+ axons (red) entering the PNG from the rostral spinal cord (green). Scale bar, 100 µm. (**e**) High-magnification confocal image showing NET+ axons (red) exiting the PNG back into the caudal spinal cord (green). Scale bar, 100 µm. (**f**–**h**) Images are from PNG-treated animals. The dotted line in each panel delineates the interface between the graft and spinal cord. (**f**) A representative low-magnification confocal image of a sagittal section with GFAP labeling (green) and TH staining (red) displaying the overall anatomy of the PNG, spinal cord, and regenerating TH+ axons. Scale bar, 500 µm. (**g**) High-magnification confocal image of the rostral cord/PNG interface boxed in f. Large densities of TH+ stained axons (red) enter the PNG (GFAP-negative area) from the rostral end of the spinal cord (green). Scale bar, 100 µm. (**h**) High-magnification confocal image of caudal PNG/cord interface boxed in f. TH+ axons (red) grow through the PNG, but fail to cross the PNG/spinal cord interface back into the caudal spinal cord (green). Scale bar, 100 µm. (**i**) Quantification of the density of TH-immunoreactive fibers found in the rostral spinal cord adjacent to the PNG, in the center of the PNG, and in the caudal spinal cord adjacent to the distal PNG. n = 6 per group. ***p < 0.001, Student’s t-test.

Our previous acute study revealed that both serotonergic (5-HT+) and tyrosine hydroxylase (TH+) axons regularly regenerated into and out of the PNG when the PNG+aFGF+ChABC paradigm was administered immediately following an injury^28^. In the chronically injured spinal cord, it was not known whether these populations of neurons retain the ability to regenerate. Thirty-two weeks following the chronic repair, the PNG+aFGF+ChABC group had robust regeneration of catecholaminergic axons, identified by being TH+ (Fig. 2a–c) and norepinephrine transporter-positive (NET+) (Fig. 2d,e). Axons could be seen entering the proximal nerve graft (Fig. 2b,d), traveling for long distances through the PNG, and crossing the PNG/spinal cord interface well into the glial fibrillary acidic protein-positive (GFAP+) host spinal cord (Fig. 2c,e). NET+ expression, which is restricted to noradrenergic neurons and is not present in neurons that release dopamine or epinephrine^30, 31^, closely mirrored TH+ staining. The PNG/saline group, which lacked aFGF and ChABC, displayed a similar density of TH+ axons as the triple combination group in the cord at the rostral graft interface (Fig. 2f,g,i). In this control group TH+ axons still frequently entered the graft, albeit at a significantly lower density than the triple combination group (Fig. 2b,g,i). However, the axons remained in the graft, never crossing the caudal interface back into the spinal cord (Fig. 2f,h,i). These results illustrate the importance of aFGF and ChABC in facilitating axonal entry into the graft, and more importantly, axonal re-entry back into the spinal cord.

**Figure 3:**
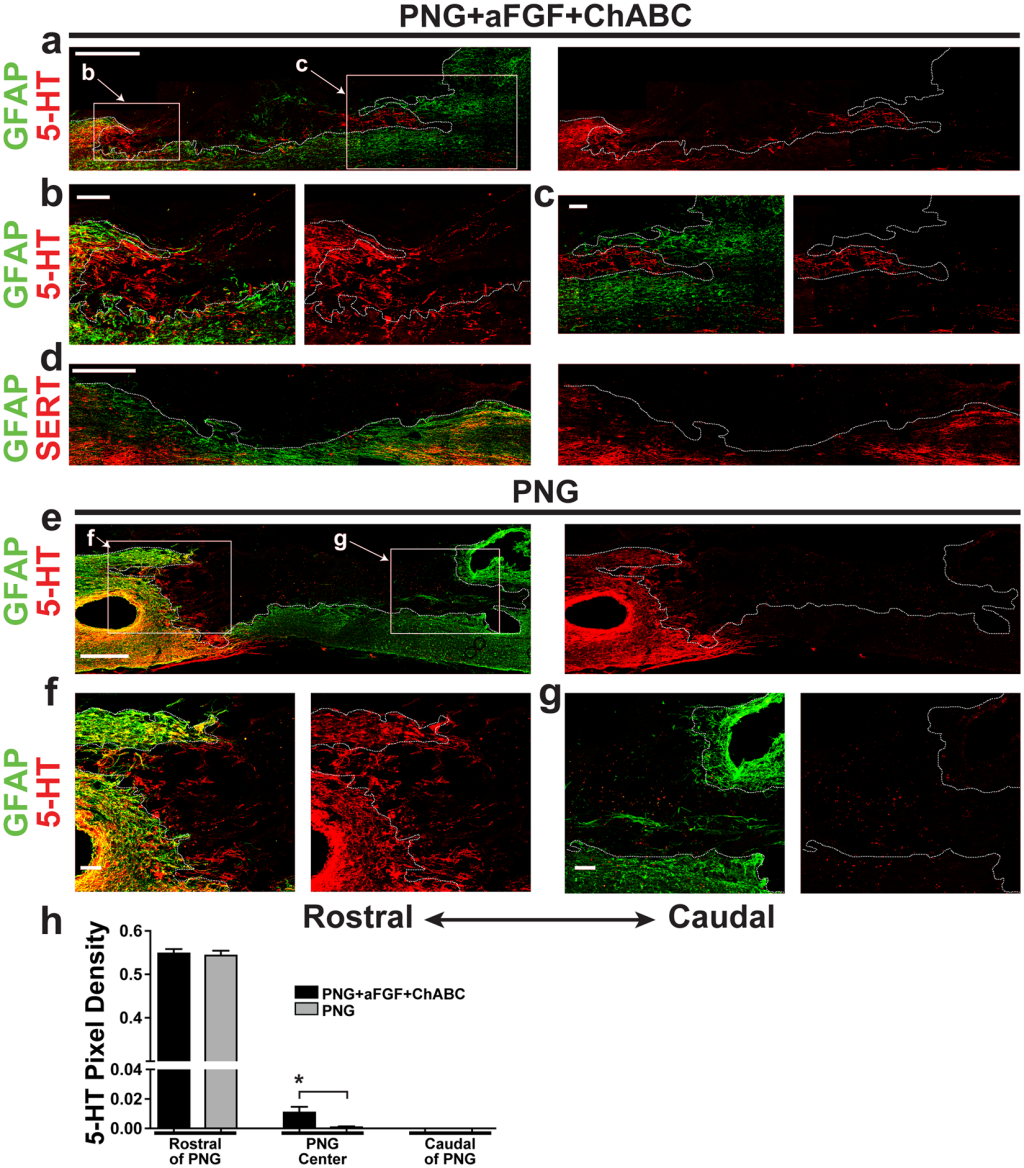
5-HT+ nerve fibers fail to regenerate in the chronically repaired cord. (**a**–**d**) Images are from PNG+aFGF+ChABC-treated animals. The dotted line in each panel delineates the interface between the graft and spinal cord. (**a**) A representative low-magnification confocal image of a sagittal section with GFAP labeling (green) and 5-HT staining (red) displaying the overall anatomy of the PNG, spinal cord, and regenerating 5-HT+ axons. Scale bar, 500 µm. (**b**) High-magnification confocal image of the rostral cord/PNG interface boxed in a. Note the small density of 5-HT+ stained axons (red) entering the PNG (GFAP-negative area) from the rostral end of the spinal cord (green). Scale bar, 100 µm. (**c**) High-magnification confocal image of the caudal PNG/cord interface boxed in a. Few 5-HT+ stained axons (red) reach the distal end of the graft, and no 5-HT axons exit the graft back into the caudal spinal cord (green). Scale bar, 100 µm. (**d**) Low-magnification confocal image of a sagittal section with GFAP labeling (green) and SERT staining (red) displaying the overall anatomy of the PNG, spinal cord, and lack of regenerating SERT+ axons. Scale bar, 500 µm. (**e**–**g**) Images are from PNG-treated animals. The dotted line in each panel delineates the interface between the graft and spinal cord. (**e**) A representative low-magnification confocal image of a sagittal section with GFAP labeling (green) and 5-HT staining (red) displaying the overall anatomy of the PNG, spinal cord, and regenerating 5-HT+ axons. Scale bar, 500 µm. (**f**) High-magnification confocal image of the rostral cord/PNG interface boxed in e. Note sparse 5-HT+ stained axons (red) entering the PNG (GFAP-negative area) from the rostral end of the spinal cord (green). Scale bar, 100 µm. (**g**) High-magnification confocal image of the caudal PNG/cord interface boxed in e. Note that no 5-HT+ stained axons (red) reach the distal end of the graft and no 5-HT+ axons exit the graft back into the caudal spinal cord (green). Scale bar, 100 µm. (**h**) Quantification of the density of 5-HT-immunoreactive fibers found in the rostral spinal cord adjacent to the PNG, in the center of the PNG, and in the caudal spinal cord adjacent to the distal PNG. n = 6 per group. *p < 0.05, Student’s t-test. Data represent mean ± S.E.M.

Conversely, in both the PNG and PNG+aFGF+ChABC groups, lower densities of serotonergic neurons, identified as 5-HT+ or serotonin transporter-positive (SERT+), regenerated into the graft (Fig. 3a,b,d–f,h). The PNG+aFGF+ChABC group displayed a modest but significant increase in 5-HT+ axons in the graft compared to the PNG group; however, we did not observe serotonergic axons re-entering the cord from the distal end of the graft (Fig. 3c,g,h). These results suggest that in the chronically injured spinal cord, catecholaminergic axons retain the ability to regenerate robustly into and out of a peripheral nerve graft, while serotonergic axons have a markedly reduced ability to regenerate in our model.

### aFGF and ChABC combined with the PNG promote astrocyte and Schwann cell integration at chronic stages

**Figure 4:**
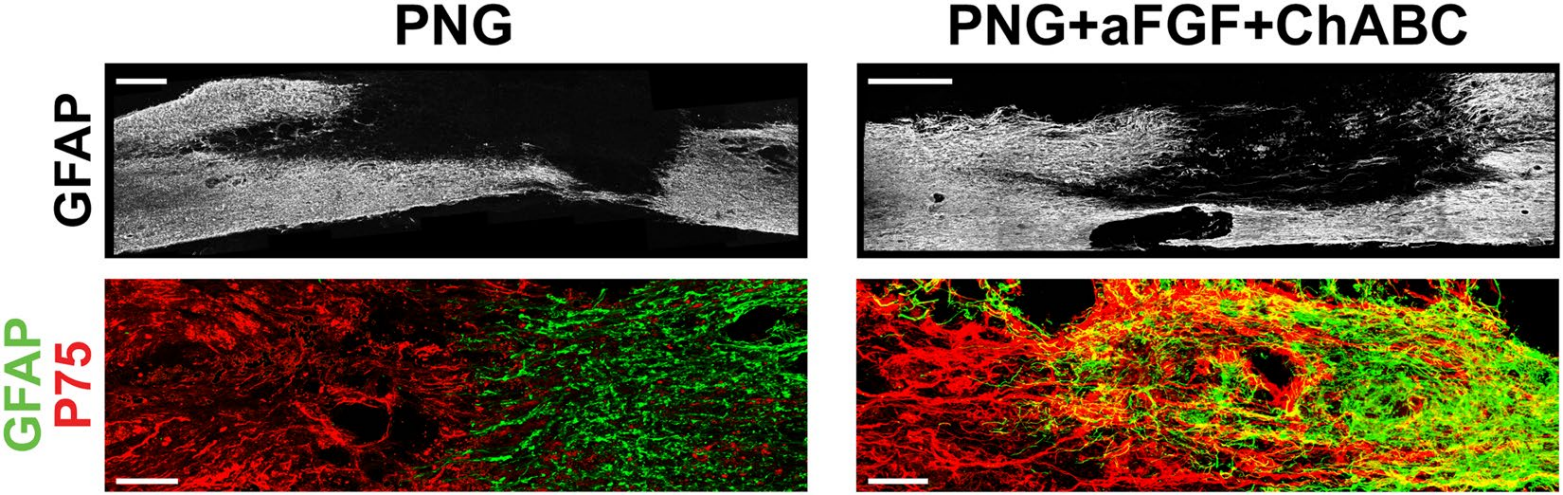
aFGF and ChABC promote astrocyte and Schwann cell integration at the interface of PNG and caudal spinal cord. Representative confocal images of sagittal sections showing PNG- (left column) or PNG+aFGF+ChABC- (right column) treated spinal cords 40 weeks post-injury. The top row shows low magnification of GFAP staining (white). Note the extensive migration of GFAP+ astrocytes throughout the PNG (GFAP-negative area) only following PNG+aFGF+ChABC treatment. Scale bar, 500 µm. Bottom row shows high magnification of PNG p75+ Schwann cells (red) and spinal cord GFAP+ astrocytes (green). Note the migration and integration of Schwann cells and astrocytes, including overlapping spatial domains (yellow), only following PNG+aFGF+ChABC treatment. Scale bar, 100 µm.

The abundance of TH+ axons growing into, but especially out of, the graft resulting from the addition of aFGF and ChABC suggested that these factors may influence how Schwann cells from the graft interact with resident reactive astrocytes in the host spinal cord. Therefore, we examined the shapes of these two populations of glial cells and the extent of overlap at the graft–host interface following PNG or PNG+aFGF+ChABC treatment. Spinal cords containing only a PNG displayed distinct boundaries between GFAP+ astrocytes and low-affinity nerve growth factor receptor, P75-positive (P75+) Schwann cells, creating a sharp border between the two cell types with no overlapping spatial domains. Astrocytes that remained close to the PNG/cord interface had migrated minimally into the graft (Fig. 4, left column). Conversely, the addition of aFGF and ChABC promoted remarkable astrocyte and Schwann cell migration and robust intermingling of the two cell types (Fig. 4, right column). Astrocytes migrated long distances throughout the entire graft and Schwann cells migrated far into the spinal cord (Fig. 4, right column). Unlike the PNG only animals, astrocytes and Schwann cells often occupied the same spatial domains, appearing as double-labeled points in confocal z-stacks (Fig. 4, right column). Furthermore, astrocyte processes, as well as Schwann cell processes, were predominantly aligned parallel to axonal processes along the longitudinal axis. These results indicate that the addition of aFGF and ChABC to the PNG/spinal cord interface enhances Schwann cell-astrocyte integration and suggest that this integration helps facilitate axon regeneration out of the graft back into the caudal spinal cord.

### The raphe, but not the locus coeruleus, increases suppressor of cytokine signaling 3 (SOCS3) expression following chronic SCI

**Figure 5:**
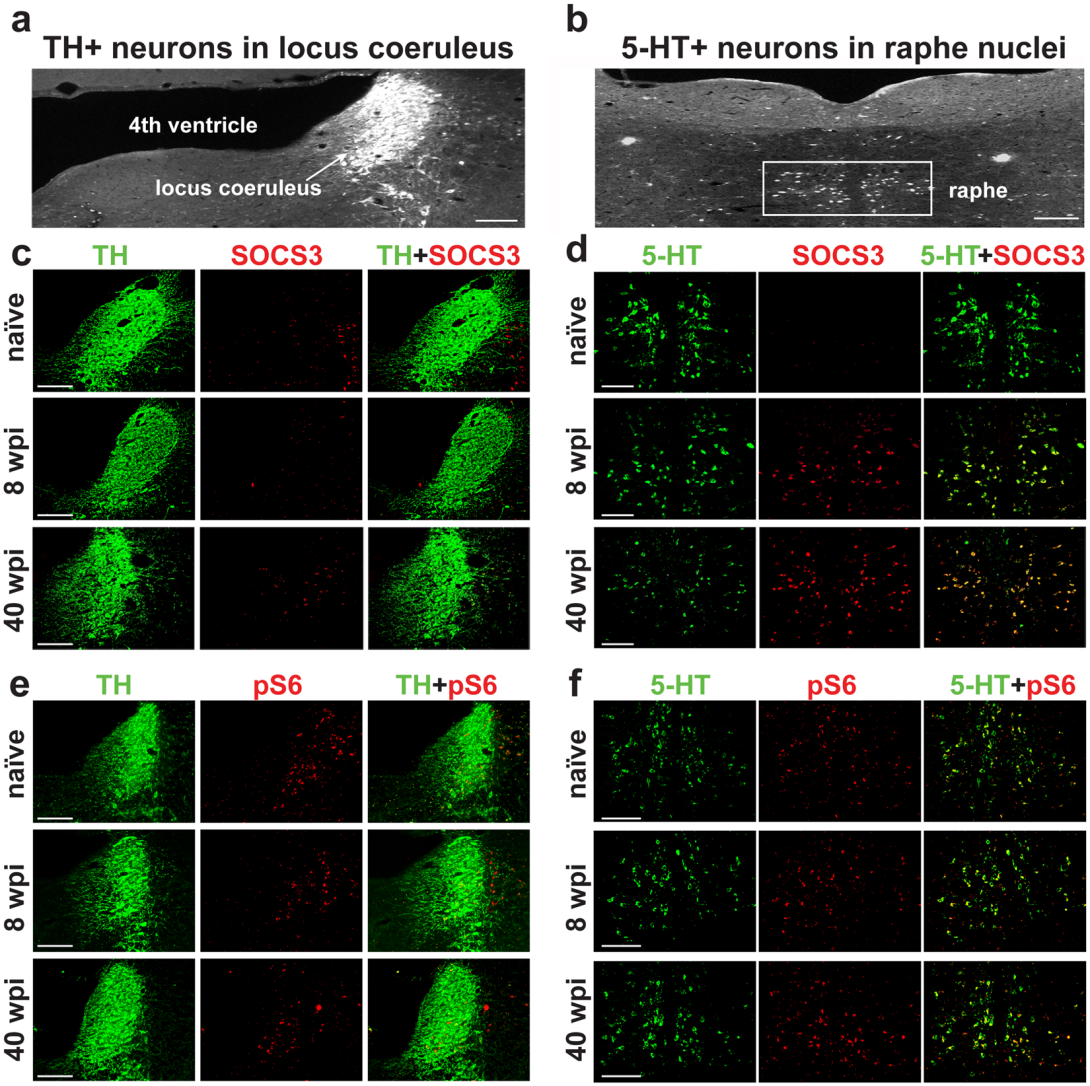
The raphe, but not the locus coeruleus, increases SOCS3 expression following chronic SCI. (**a**) Fluorescent image of the brainstem with TH staining illuminating the locus coeruleus. (**b**) Fluorescent image of the brainstem with 5-HT staining illuminating the raphe. (**c**) Fluorescent images of the locus coeruleus with TH (green) and SOCS3 (red) staining in naïve cord, eight weeks post SCI, or 40 weeks post SCI treated with PNG+aFGF+ChABC. (**d**) Fluorescent images of the raphe with 5-HT (green) and SOCS3 (red) staining in naïve cord, eight weeks post SCI, or 40 weeks post SCI treated with PNG+aFGF+ChABC. (**e**) Fluorescent images of the locus coeruleus with TH (green) and pS6 (red) staining in naïve cord, eight weeks post SCI, or 40 weeks post SCI treated with PNG+aFGF+ChABC. (**f**) Fluorescent images of the raphe with 5-HT (green) and pS6 (red) staining in naïve cord, eight weeks post SCI, or 40 weeks post SCI treated with PNG+aFGF+ChABC. Scale bar, 100 µm.

Chronically injured TH+, but not 5-HT+, neurons retain the ability to robustly regenerate in our triple combination therapy, suggesting that differences exist in how these two neuronal populations respond to contusive injury over time. We, therefore, examined the locus coeruleus and raphe, regions of the brainstem respectively housing TH+ (Fig. 5a) and 5-HT+ (Fig. 5b) somata, for expression changes of intrinsic factors known to control axon re-growth. Prior to the injury, both the locus coeruleus and raphe expressed low levels of SOCS3 (Fig. 5c,d), an important negative regulator of injury-induced growth-promoting factors^32^. By eight weeks post injury (wpi), at the time of the second repair surgery, a strong and persisting upregulation of SOCS3 was observed in the raphe, which was maintained until the end of the study at 40 wpi (Fig. 5d). On the other hand, SOCS3 expression in the locus coeruleus remained low at all observed time points (Fig. 5c). SOCS3 expression at 6 months post injury without a second surgical intervention was elevated in the raphe but not the locus coeruleus, similar to what we observed following the triple combinatorial treatment, suggesting that neither the second surgery nor the treatment affect SOCS3 expression (data not shown). Several studies have also identified that activation of the mammalian target of rapamycin/phosphatase and tensin homolog (mTOR/PTEN) pathway inhibits axon regeneration^33, 34^. We found no evidence of mTOR/PTEN activation as uninjured rats displayed similar levels of phosphorylated S6 (pS6) in the locus coeruleus and raphe, which did not change noticeably in their injured counterparts at 8 wpi or 40 wpi (Fig. 5e,f). This data suggests that SOCS3 expression in the locus coeruleus and raphe negatively correlates with the growth potential of these two systems and may contribute to regeneration or sprouting failure of chronically injured 5-HT+ neurons in the spinal cord.

### PNG+aFGF+ChABC increases lumbar TH+ axons around the anterior horn of the spinal cord

**Figure 6:**
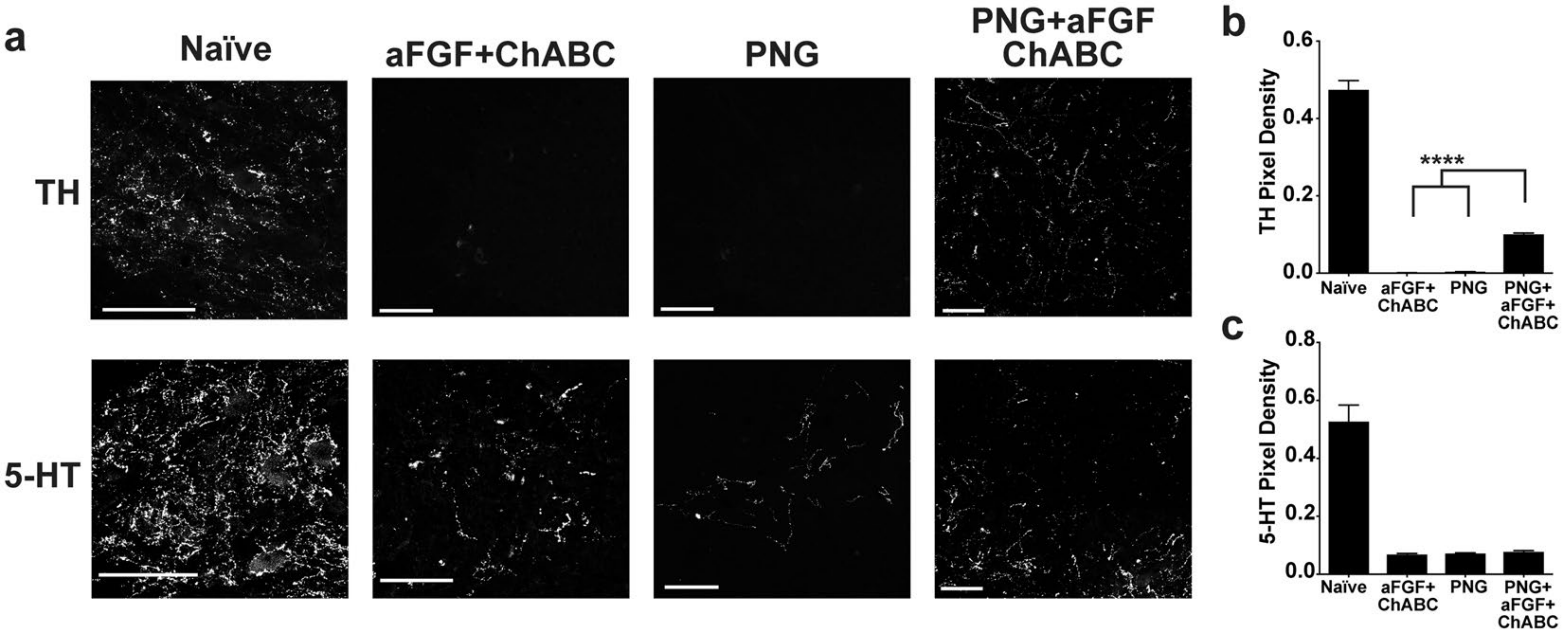
PNG+aFGF+ChABC increases lumbar TH+ axons around the anterior horn. (**a**) Representative confocal images of TH+ (top row) or 5-HT+ (bottom row) stained transverse sections in the ventral grey horn of the lumbar spinal cord 40 weeks post-injury. Scale bar, 100 µm. (**b**,**c**) Quantification of the density of TH+ (**b**) and 5-HT+ (**c**) immunoreactivity in the ventral grey horn of the lumbar spinal cord. n = 6 per group except naive n = 4. ****p < 0.0001. One-way ANOVA, Fisher’s Least Significant Difference post-hoc test. Data represent mean ± S.E.M.

The caudal spinal cord houses second order efferent neurons and interneurons critical for the control of locomotion and micturition. Noradrenergic and serotonergic neurons have been shown to be important modulators of both locomotor^35, 36^ and bladder^37, 38^ function. We next examined the lumber cord for the presence of 5-HT+ and TH+ axons within the ventral horn. All injured groups showed a decrease in TH+ and 5-HT+ axon densities compared to the uninjured naive cord; however, the PNG+aFGF+ChABC-treated animals displayed a relative increase in TH+ density when compared to the PNG or aFGF+ChABC treatment groups (Fig. 6a,b). All injured groups displayed similar low 5-HT+ levels regardless of treatment conditions (Fig. 6a,c). These results are consistent with the observed TH+ axon regeneration and the lack of 5-HT+ axon regeneration at the thoracic injury site following PNG+aFGF+ChABC treatment (Figs 2 and 3) and suggest that in the chronic state of SCI the triple combination treatment may promote long distance regeneration or sprouting of spared axons distal from the injury site in certain neural populations.

### PNG+aFGF+ChABC maintains locomotor skills and improves lower urinary tract recovery after chronic injury

**Figure 7:**
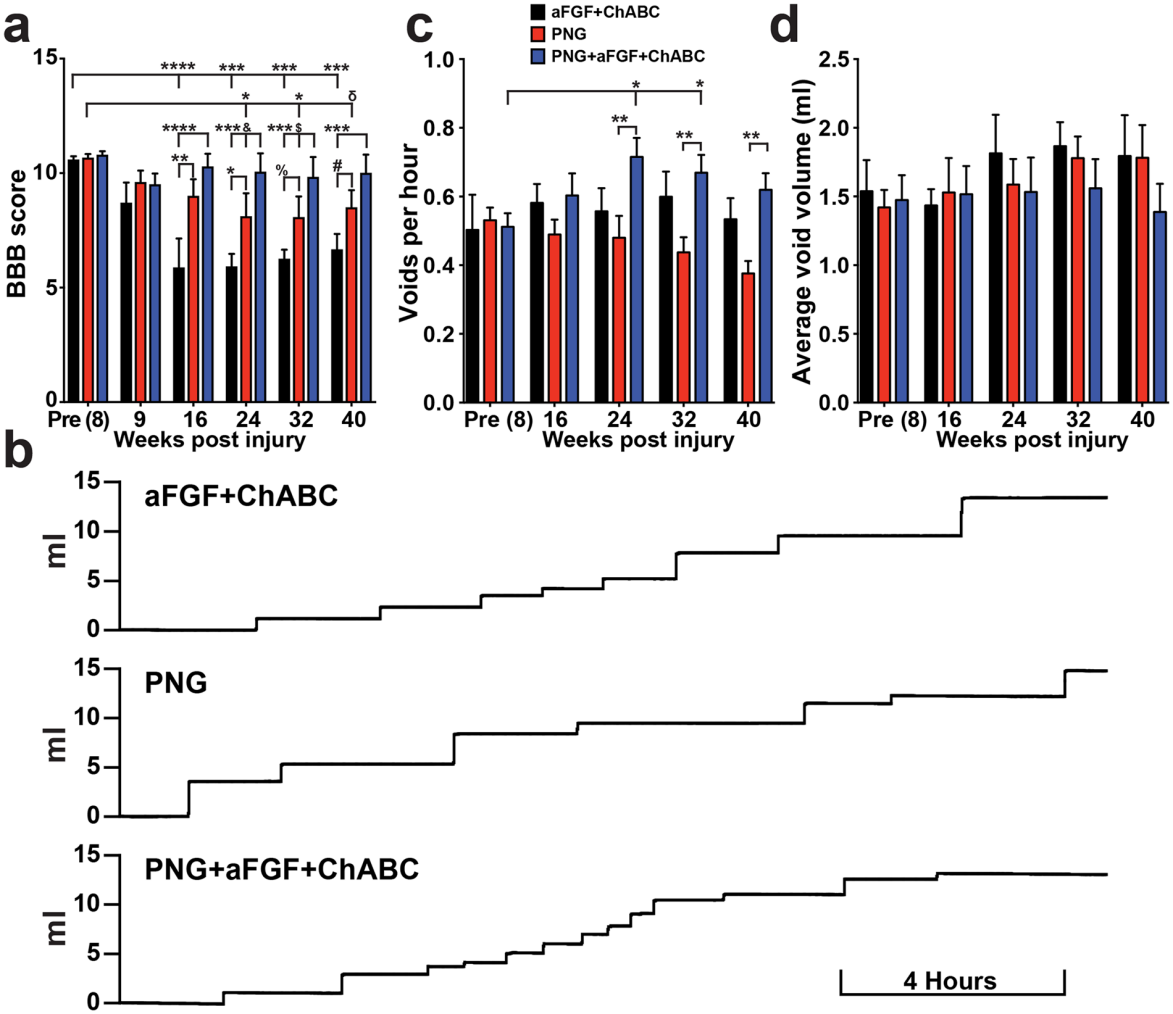
PNG+aFGF+ChABC treatment maintains locomotor and improves micturition patterns. (**a**) Locomotor BBB score of rats prior to the repair surgery (Pre (8)) and following the repair surgery (weeks 9–40). n = 6 per group except at week 9 n = 5 for PNG and aFGF+ChABC. Between groups: ^&^p = 0.0640, ^$^p = 0.0931, ^%^p = 0.0816, ^#^p = 0.0794, *p < 0.05, **p < 0.01, ***p < 0.001, ****p < 0.0001 Two-way repeated measures ANOVA Fisher’s Least Significant Difference post-hoc test. Within group ^δ^p = 0.0582, *p < 0.05, ***p < 0.001, ****p < 0.0001 One-way ANOVA, Fisher’s Least Significant Difference post-hoc test. (**b**) Representative smoothed metabolic cage traces 40 weeks after injury. (**c**,**d**) Metabolic cage quantification of average void frequency (**c**) and void volume (**d**). n = 6 per group. Between groups: **p < 0.01. Two-way repeated measures ANOVA, Fisher’s Least Significant Difference post-hoc test. Within group *p < 0.05 One-way ANOVA, Fisher’s Least Significant Difference post-hoc test. Data represent mean ± S.E.M.

SCI often results in the permanent decline of locomotor and lower urinary tract function. We tracked locomotor recovery prior to and following the repair surgery using the Basso, Beattie and Bresnahan (BBB) scale^39^. Eight weeks post-injury and prior to the repair surgery, all animals were able to weight-support step, corresponding to a BBB score of 10–11 (Fig. 7a). Following the repair surgery, the PNG and aFGF+ChABC control groups lost the ability to weight-support step (BBB < 10). However, animals treated with PNG+aFGF+ChABC maintained the ability to occasionally weight-support step (Fig. 7a). PNG+aFGF+ChABC treatment maintained significantly better BBB walking scores than the aFGF+ChABC and PNG treatment groups beginning at about eight weeks or 16 weeks post-repair surgery, respectively, and continuing until the end of the study (Fig. 7a). These results suggest that PNG+aFGF+ChABC treatment did not lead to robust recovery of walking, but rather attenuated the decline in locomotor ability that develops following an invasive repair surgery.

**Figure 8:**
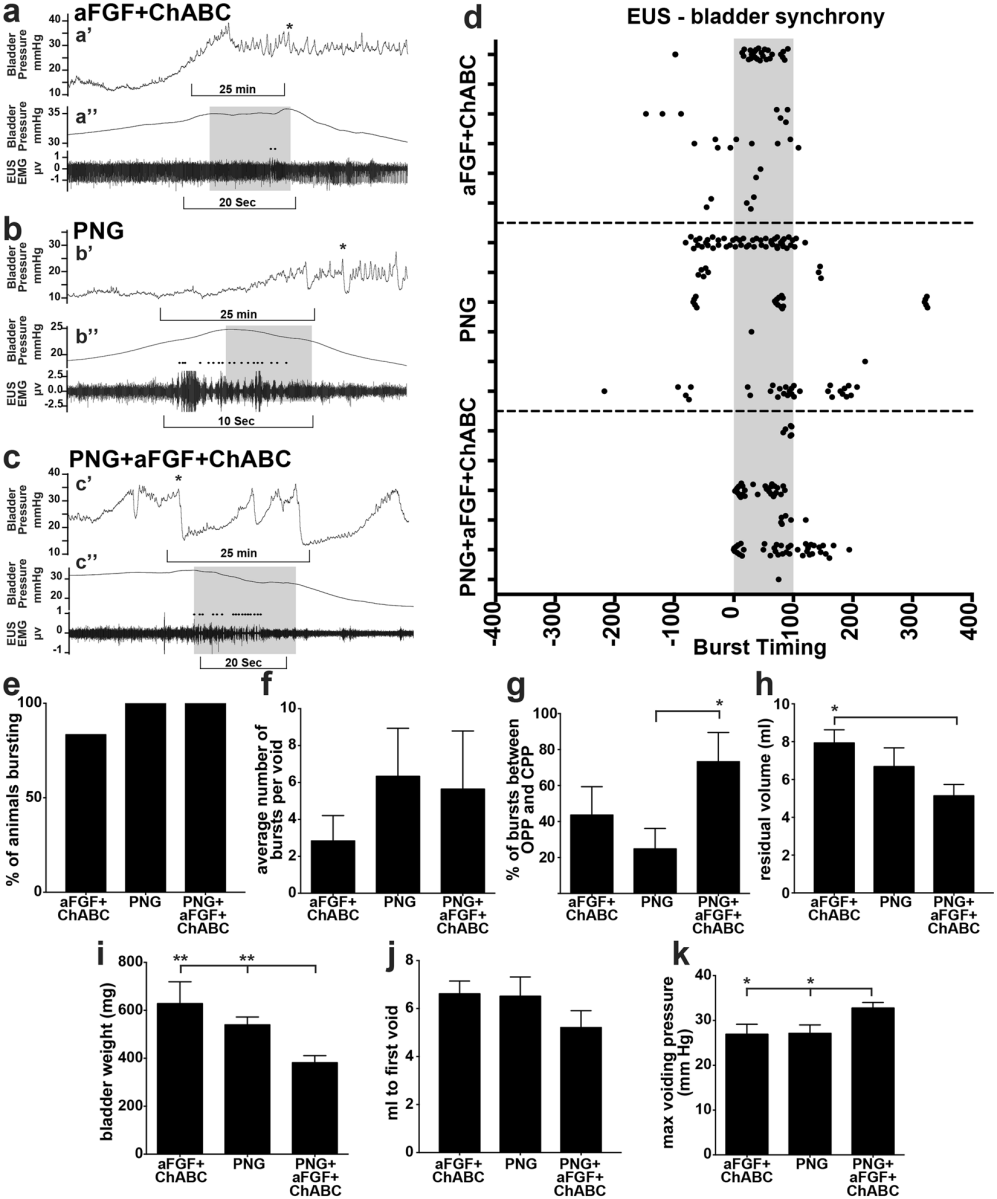
PNG+aFGF+ChABC treatment improves urodynamic recovery. (**a**–**c**) Representative urodynamic recordings at 40 weeks after SCI. (a’-c’) Compressed view of bladder pressure recordings. Asterisks mark expanded void shown below. (a”-c”) Expanded time points from a’-c’. Grey box denotes the time between opening peak pressure (OPP) and closing peak pressure (CPP). Black dots denote EUS bursting. (**d**) Scatter plot of EUS bursting over three void cycles in relation to bladder contractions for individual animals. Grey box denotes the time interval between OPP and CPP (0–100%). (**e**) Percentage of animals displaying EUS bursting. (**f**) Average number of EUS bursts per void. (**g**) Percentage of EUS bursts between OPP and CPP of bladder contractions. (**h**) Average residual bladder volume following a void. (**i**) Bladder weight 40 weeks post-injury. (**j**) The bladder volume needed to initiate the first void. (**k**) The maximal pressure reached during a void. n = 6 per group. *p < 0.05, **p < 0.01. One-way ANOVA, Fisher’s Least Significant Difference post-hoc test. Data represent mean ± S.E.M.

Lower urinary tract dysfunction is a major concern among the spinal cord-injured population^40^. In humans, SCI-induced urinary retention is treated by catheterization, which severely lowers the quality of life and contributes to urinary tract infections. Following SCI, micturition patterns in rats shift to fewer and larger voids, quantified using metabolic cage recordings (Fig. 7b). Prior to the repair surgery, all groups displayed a similar void frequency and volume (Fig. 7c,d). Following the repair surgery, PNG+aFGF+ChABC treatment significantly improved void frequency beyond that of the pre-repair rate and also beyond that in the two control groups (Fig. 7b,c). No changes were observed in void volume (Fig. 7b,d). Next, we investigated the coordinated recovery of muscles controlling micturition at 40 weeks post-injury using terminal urodynamic analyses. During a normal voiding event in a non-spinalized rat, the external urethral sphincter (EUS) phasically bursts in coordination with a detrusor contraction, expressing urine out of the bladder through the urethra. Most EUS bursts occur between the opening peak pressure and closing peak pressure of a bladder contraction, outlined in grey boxes in Fig. 8a–c. Following injury, coordination between the EUS and detrusor is lost, resulting in bladder contractions against a closed urethra and inappropriately timed EUS bursting. All but one rat in the aFGF+ChABC group retained the basic ability to burst (Fig. 8d,e). PNG- and PNG+aFGF+ChABC-treated animals displayed more bursts per void than the aFGF+ChABC group (Fig. 8d,f). However, only in the PNG+aFGF+ChABC-treated animals were the majority of bursts synchronized between the opening and closing peak pressure of a bladder contraction (Fig. 8d,g). Improved coordination led to an increased voiding efficiency and decreased residual volume (Fig. 8h). Following SCI, the bladder enlarges and distends to accommodate a larger capacity of urine due to inefficient voiding. The bladders of animals treated with PNG+aFGF+ChABC were smaller (Fig. 8i), elicited a void at a smaller volume (Fig. 8j), and generated a greater pressure difference during the void, indicating a smaller and stronger bladder. These results suggest that PNG+aFGF+ChABC treatment delivered during the chronic stage after SCI improves bladder-EUS synchrony, resulting in voids that are more efficient and a healthier bladder.

## Discussion

Substantial barriers exist to inhibit axonal regeneration that could lead to functional recovery after chronic SCI. The lesion environment is densely scarred and laden with inhibitors^3^, the surviving neurons decline in their intrinsic ability to grow^6, 7^, and persistent inflammation hinders proper wound healing^41^. Many therapies developed to treat acute stages of SCI, such as immune modulation^42^ or inhibition of scar formation^43^, will not translate to injuries in more chronic states. While we previously showed that our triple combination repair strategy consisting of PNG+aFGF+ChABC promoted the regeneration of acutely lesioned axons into, and most importantly, out of grafts^28, 44^, it was unknown whether this strategy might also be efficacious in the chronically injured state. In the current study, we utilized a contusion injury, the most common type of SCI^29^, rather than a full transection injury used in the acute models. We report here, for the first time, that treatment of a chronically contused spinal cord with PNG+aFGF+ChABC promotes significant integration between PNG Schwann cells and reactive astroglia of the cord which, in turn, allows noradrenergic but not serotonergic axons to regenerate. Additionally, this treatment lead to functional improvements, especially in bladder physiology.

It has long been known that axons can readily grow into a PNG^45–47^; however, almost no axons will cross from the PNG through a glial scar back into the CNS without additional manipulations^46^. We addressed this regeneration shortfall through the removal of inhibitory CSPGs at the graft/host interface, by supplying trophic support to promote lengthy axonal growth, and by enhancing integration between the graft and spinal cord glia.

### ChABC and aFGF’s contribution to regeneration

ChABC digestion of CSPGs enhances axonal regeneration and sprouting in a variety of injury models^8, 18, 19, 48, 49^. The addition of ChABC to a PNG repair model resulted in significantly more axons crossing the graft/cord interface and improved functional recovery^19, 28^. Axonal growth is also enhanced through aFGF stimulation. FGF has been shown to promote long-distance, unbranched axon regrowth^50, 51^, in contrast to other neurotrophins such as NGF, BDNF, or neurotrophin-3, which promote a more branched pattern of growth that may hinder rectilinear elongation^52^. However, since a contused spinal cord contains spared fibers, other possible mechanisms beyond regeneration from injured axons, such as sprouting of uninjured tracts^53^, activation of redundant or latent pathways^54^, and alterations to postsynaptic receptors or activation thresholds^55^, cannot be excluded from contributing to recovery.

### ChABC and aFGF’s contribution to the graft/cord interface

Our results have also shown that the addition of aFGF and ChABC to the PNG in a chronically injured cord strongly promotes astrocyte orientation towards and migration well into the Schwann cell-laden graft, and vice versa. A characteristic feature of successful tissue repair between the PNG and CNS compartment is the integration of donor and host glia. Bridge building by astrocytes, even when they are in a fully reactive state, has been described as an anatomical predictor of axon regeneration across lesions^56–62^. Several studies with successful axon regeneration into or through peripheral nerve repair grafts exhibit integrating migration of astrocytes and Schwann cells at the graft/cord interface^58, 63–67^, whereas a lack of integration is associated with regeneration failure^58, 67, 68^. In our chronic contusion model, astrocyte/Schwann cell interactions were surprisingly enhanced even beyond that which occurs at acute stages by the addition of aFGF and ChABC. In mammals, aFGF has frequently been shown to reduce axonal die-back, to promote regeneration through a graft, and to reduce astrocyte reactivity^24, 25, 27, 69, 70^. Interestingly, FGF signaling is also crucial for the formation of astrocyte bridges, upon which regenerating axons cross a transected zebrafish spinal cord^26^. Both zebrafish and human astrocytes undergo a dramatic transformation from a stellate shape to a bipolar, radial morphology upon aFGF stimulation^26^, bearing a remarkable resemblance to the long, fingerlike astrocytic projections in successful grafts observed here and previously^28, 66^. Failure to include aFGF in a PNG or Schwann cell graft significantly diminishes the abundance of regenerating axons found within the graft^28, 71^. While more evidence exists for the role of aFGF in graft/cord interface integration, ChABC digestion of CSPGs may also contribute to the improved interface by reducing basal lamina formation thereby facilitating the migration of Schwann cells into the host spinal cord^20^, as well as aligning and allowing for reverse migration of astrocytes towards and into the graft site^21^. While we did not investigate the effects of ChABC or aFGF individually in this current chronic study, our previous acute study suggests that leaving out either aFGF or ChABC severely diminishes the prevalence of regenerating axons and disrupts the PNG/cord interface^28^.

### Removal of the glial scar

Following contusion injury, the scar which is comprised of multiple layers of densely packed and highly reactive astrocytes lined by a population of collagen producing reactive pericytes forms a smooth surface facing the cavity^72–74^. Removal of the scar prior to transplantation has been reported by others to improve graft/cord integration^75^ and to enhance regeneration^15, 76^. In this study, in order to permit graft apposition with the cord parenchyma, we gently extracted the mature scar tissue lining the injury cavity. Scar removal may promote an acute-injury response and/or the removal of physical and biochemical hindrances, thereby enhancing integration. Thus, it is possible that, while minimally invasive, removal of this layer helped to trigger plastic changes in chronically reactive astrocytes lying slightly deeper which, in turn, may have allowed their enhanced integration with Schwann cells. However, the molecular mechanisms that lead to such far-ranging mixing of the two cell types in the chronic state, and how aFGF+ChABC facilitates this integration, remains to be elucidated. Further, it is unknown if aFGF+ChABC is sufficient for integration in the absence of scar removal.

### The regeneration potential of chronically injured 5-HT+ and TH+ axons

A major observation of this study is the contrasting regeneration potential of different chronically injured neuronal populations. When our triple combination treatment was applied immediately following injury, a time at which SOCS3 expression is low in both the raphe and locus coeruleus, both 5-HT+ and TH+ axons robustly regenerated through the PNG and back into the distal cord^28^. However, in the chronic state, only TH+ axons regenerated in appreciable densities, whereas 5-HT+ neurons, which strongly increased SOCS3 expression, failed to re-grow. Others have reported limited regeneration of serotonergic axons in the chronic injury^14, 66, 77, 78^. Similar to our observation, these studies described 5-HT axons growing into a graft or lesion epicenter, but did not show regeneration out of the graft or into the distal cord. The decrease in regenerative potential appears to be time-dependent, as 5-HT axons are still capable of regeneration through a fetal spinal transplant in the sub-acute stage at two weeks post-injury^79^. The contribution of SOCS3 expression to, and how it is impacting, regeneration failure in the chronic injury state remains to be determined experimentally. Indeed, SOCS3 may only be one among several factors at play; including upregulation of other intrinsic growth-regulatory genes^80^, as well as increased expression or signaling of receptors that recognize extrinsic inhibitory molecules, such as myelin or CSPGs^81, 82^. However, the lack of expression changes observed in mTOR/PTEN indicates that the SOCS3 pathway may play a more important role in regulating nerve regeneration from brainstem neurons. Further investigation is also needed to determine whether other populations of acutely regenerating axons, such as propriospinal or dorsal column axons, retain or lose their regenerative capacity over time.

### Bladder recovery following PNG+aFGF+ChABC treatment

A leading factor affecting the quality of life of individuals living with SCI is bladder control^40^. We observed significant, albeit modest, improvements in lower urinary tract recovery with PNG+aFGF+ChABC treatment that were especially correlated with improved bursting synchrony between the EUS and bladder detrusor. While EUS bursting activity in general is known to be modulated by 5-HT in the lumbosacral spinal cord^38, 83, 84^, we observed very little 5-HT regeneration or sprouting in the thoracic or lumbar segments in any of the groups. This could be the likely explanation for the consistently low levels of bursting for most animals regardless of treatment. Nonetheless, as a group, PNG+aFGF+ChABC animals displayed greater EUS-bladder synchrony, lower residual volume, smaller overall bladders, and a greater frequency of voiding, suggesting that the TH+ system or other unidentified tracts also play a role in the recovery of lower urinary tract function, as we have observed in the acute treatment paradigm^28, 44^. While physiological recovery was correlated to regeneration, we cannot discount how the PNG+aFGF+ChABC treatment may be acting on intact tracts to promote compensatory sprouting and the return of function.

The current study provides an experimental framework for the treatment of a long-standing contusive SCI. The transplantation of peripheral nerve autografts supplemented with aFGF and ChABC promoted axonal regeneration by chronically injured neurons and provided an avenue to improve locomotor and bladder function. Future challenges will be to target neuronal populations that fail to grow in the chronic state by either creating even more favorable environments^85^ or by targeting intrinsic growth programs such as SOCS3. However, it is evident by this work and others that a combination of approaches targeting many aspects of SCI pathology will most likely be needed.

## Methods

### Study design

Thirty-three Sprague-Dawley rats (225–250 g) underwent a 250 kdyne contusion at vertebral T8. Eight weeks post-injury, rats with a BBB score of 10 or greater (range 10–11) were randomly assigned into three groups (n = 24) and underwent a second surgery: PNG, aFGF+ChABC, or PNG+aFGF+ChABC. Animals were then observed for 32 additional weeks. During this time, any rats developing health complications were removed from the study (2 in each group). Each rat reaching the study endpoint was included in the data analysis. The final group size is n = 6 per group. Locomotor assessments were evaluated with the BBB open field locomotor test performed monthly. Bladder assessments were performed by metabolic cage analysis prior to the second surgery and monthly thereafter. Cystometric analysis with EUS EMG recording was performed 40 weeks post-injury, followed by sacrifice and histological investigation in both brain and spinal cord. Another cohort of 4 naïve animals were also sacrificed for histological comparisons.

### Contusive spinal cord surgery, multiple peripheral nerve segment transplantation, and aFGF+ ChABC injection

All sterile surgical procedures were performed in strict accordance with the recommendations in the Guide for the Care and Use of Laboratory Animals of the National Institutes of Health. The protocol was approved by the Institutional Care and Use Committee of both the Case Western Reserve University animal resource center and the Cleveland Clinic. Adult female Sprague-Dawley rats (225–250 g) were obtained from Harlan/Envigo and acclimated to the animal resource center, behavior analysis chambers, and handlers. Rats were intraperitoneally injected with ketamine (60 mg/kg) and xylazine (10 mg/kg). The musculature was cut from T7-T9 and the dorsal surface of T8 was exposed by laminectomy. The vertebral column was stabilized by clamping the T7 and T9 vertebral bodies with forceps fixed to the base of the Infinite Horizon Impact Device. The animals were situated on the platform and the 2.5mm stainless steel impactor tip was positioned over the midpoint of T8 and impacted with 250 kDyne force. The overlying musculature was sutured closed and the skin was closed using wound clips. The animals were treated with Marcaine at the incision site. A force/displacement graph was used to monitor impact consistency and any animals that exhibited an abnormal impact graph or greater than 10% deviation from 250kDyne were immediately excluded from the study (n = 0). Manual bladder expression was performed 2–3 times daily for two-three weeks until a voiding reflex returned. Eight weeks following the contusive SCI, a second surgery was performed under 2% isoflurane mixed with oxygen anesthesia. The thoracic spinal cord was re-exposed and the dorsal spinal cord was incised to expose the underlying cavity. Scar tissue was gently teased out of the cavity. Two μl (0.5 μl at the rostral and caudal lesion, and 0.5 µl on the lateral sides of the lesion) of saline or 1:1 ChABC (1U/ml) & aFGF (10 μg) mixture was injected into the spinal cord via a Nanoject II (Drummond Scientific Company). For the PNG and PNG+aFGF+ChABC groups, the intercostal nerves were collected, soaked in ChABC (1U/ml) or saline for 30 min, and then two to four PNG bridges were constructed from segments spanning the lesion. The graft was supported using an aFGF/ChABC/fibrin glue. Monofilament sutures (4-0) were used to close the skin and musculature. Bladders were manually expressed twice per day until a voiding reflex returned.

### Behavioral analyses of locomotion and bladder function

#### BBB open field locomotor assessment

Blinded observers conducted all behavioral analyses. BBB score was performed as described previously^39^. Each animal was tested prior to the second surgery and monthly thereafter, through week 40.

#### Metabolic cage micturition

Prior to the second surgery and monthly thereafter, animals were placed overnight in a metabolic cage (Braintree Scientific) with access to ample food and water for the measurement of voiding patterns. Urine was collected on a force transducer and strain gauge (Grass Technologies) and plotted in Spike2 (Cambridge Electrical Design, sampled at 20HZ). Micturition pattern analysis included the frequency of voiding in a 16-hour period and the volume per void. The total volume of expelled urine was not included because of variation in water intake between individual animals.

#### Urodynamics

Terminal urodynamic recordings were performed as described previously^28^. Briefly, rats were anesthetized at 40 weeks post-SCI with 0.8 g/kg urethane delivered subcutaneously. A polyethylene-50 catheter was carefully inserted through the urethra into the bladder for the delivery of saline. Fine-wire electrodes (0.003″ diameter Teflon-insulated silver wire; A-M Systems) were inserted percutaneously via the vagina on both sides of the urethra to monitor EUS electromyography (EMG) activity. The electrodes were connected to a preamplifier (HZP; Grass-Technologies), which was connected to an amplifier (QP511, Grass-Technologies) with high- and low-pass frequency filters at 30 Hz and 3 kHz and a recording system (Power 1401, Spike2; Cambridge Electronic Design) at a sampling frequency of 10 kHz. Continuous cystometrograms (CMGs) were collected using constant infusion (6 ml/hr) of room temperature saline (Aladdin-1000 single syringe infusion pump; World Precision Instruments) through the catheter into the bladder to elicit repetitive voids. The bladder pressure was recorded via the same catheter used for saline infusion, using a pressure transducer (P23XL, Grass Technologies) connected to strain gauge (P11T, Grass Technologies), which fed into the recording system and sampled at 2 kHz. Quantification of bursting was completed using Spike2 and EXCEL (Microsoft). The timestamp of the opening peak pressure (OPP) and closing peak pressure (CPP) were measured and assigned a value from 0 to 100 (0–100%), respectively, to normalize the length of time between the OPP and CPP between animals and when bursting occurred. Bursting timestamp was then marked via custom Spike2 scripts and given a percentage value as to when the burst occurred based on the corresponding OPP and CPP timestamps.

#### Perfusion and immunohistochemistry

Rats were transcardially perfused with ice-cold 4% paraformaldehyde in PBS and the spinal cords and bladders were dissected. The tissue was post-fixed in 4% paraformaldehyde overnight at 4 °C and then cryoprotected with 30% sucrose. Spinal cords and brains were then frozen in OCT mounting media and sectioned on a Hacker cryostat at a thickness of 30 µm. Free-floating sections were washed three times with PBS followed by blocking in 3% normal goat serum (NGS) or normal donkey serum (NDS) and 1% bovine serum albumin (BSA) in PBS. 0.25% Triton-X was added to the blocking buffer depending on the antigen used. Following blocking, sections were incubated in primary antibody diluted in blocking buffer overnight at room temperature. Primary antibodies used were anti-TH polyclonal antibody (1:1000 dilution; Protos Biotechnology), anti-5-HT polyclonal antibody (1:1500 dilution; DiaSorin), anti-GFAP antibody (1: 4000 dilution; EMD Millipore), anti-SOCS3 antibody (1:1000 dilution; Abcam), anti-pS6 antibody (1:7500 dilution; Cell Signaling Technology), anti-NET antibody (1:500; EMD Millipore), anti-SERT antibody (1:1000 dilution; Calbiochem), or anti-p75 antibody (1:1000 dilution; Abcam). After 3 rinses (10 mins per time) in PBS, sections were incubated with species-appropriate secondary antisera conjugated with Alexa Fluor 594 or Alexa Fluor 488 (Invitrogen/Molecular Probes) for 90 min, washed for 3 times (10 mins per time), mounted on the slides, and then coverslipped with Vectashield (Vector Laboratories Inc., Burlingame, CA, USA). All sections were examined using a Leica DM5000 fluorescent microscope with deconvolution, and the distribution of TH and 5-HT fibers was analyzed in multiple parallel sections. Further images were collected using a Carl Zeiss LSM 510META confocal microscope or Leica SP8 confocal microscope.

#### Pixel density analysis

Color images of interest obtained via fluorescent microscopy under identical exposure settings. Images were then converted to a grayscale prior to pixel intensity analysis. Grayscale follows an index between 0–255 (black-white) with an index of 0 and 255 corresponding to background and maximum expression under fixed microscopy settings, respectively. A custom MATLAB program was written to normalize the image background to an index of 0 (black), and calculate the area of positive expression as the number of pixels exceeding the background threshold index within the image frame. The number of positive pixels was then divided by the total number of pixels to obtain the pixel density.

#### Data analysis

All statistical analyses were performed using Prism (Graphpad) and were considered significant when p < 0.05. All results are presented as mean ± s.e.m. D’Agostino–Pearson and Shapiro–Wilk tests were first performed to determine normality of data. For TH+ and 5-HT+ regeneration in the thoracic cord, a 2-tailed Student’s T-test was performed. For BBB score and metabolic cage analyses, two-way repeated measures ANOVAs were performed to compare between all injured groups from weeks 16–40. Post-hoc analyses between different treatment groups at individual time points were performed with Fisher’s Least Significant Difference post-hoc test. To compare within a group, an ANOVA was performed with Fisher’s Least Significant Difference post-hoc test to compare pre-repair surgery values with post-repair surgery values. Urodynamic data and lumbar immunoreactivity were evaluated via ANOVAs between injured groups with Fisher’s Least Significant Difference post-hoc test.
